# Cyberdelics in context: On the prospects and challenges of mind-manifesting technologies

**DOI:** 10.3389/fpsyg.2022.1073235

**Published:** 2023-01-13

**Authors:** Ido Hartogsohn

**Affiliations:** The Graduate Program in Science, Technology and Society, The Interdisciplinary Studies Unit, Bar-Ilan University, Ramat Gan, Israel

**Keywords:** psychedelics, cyberdelics, virtual reality, neurofeedback, imaginaries, consumerism

## Abstract

The concept of cyberdelics emerged in the 1980s and 1990s as an umbrella term denoting the nexus connecting cybernetic (digital) technologies and psychedelic (mind manifesting) drugs. Cyberdelic technologies, in particular the then newly emerging field of virtual reality, were touted by psychedelic cultural icons including Timothy Leary and Terence McKenna as auguring a new era of digital mind-expansion where psychedelic experiences will be recreated online inside virtual worlds. Cyberdelic culture waned in the 2000s. However, recent years have seen the return of the cyberdelic imaginary, following on the heels of a psychedelic resurgence and a renewed interest in virtual reality technologies and their use in therapy. Cyberdelic advocates speak of the necessity of creating transformative technologies that steer humanity away from mindless consumerism and distractedness, and towards expanded states of awe, presence, and transcendence. Nevertheless, much like psychedelics, cyberdelic technologies are seen as running against the grain of current sociocultural arrangements and economic models which threaten to quell their transformative potential. Research on psychedelics within the humanities over the past decade has emphasized the role of cultural set and setting: the significance of the cultural embeddedness of these psychoactive agents and the dependence of their effects on surrounding sociocultural conditions. Building on the notion of information technologies as mind-manifesting technologies, this paper sets out to consider what psychedelics can teach us about cyberdelics: how the principles of set and setting and current discussions within the psychedelic humanities can inform our understanding of the resurgence of interest in cyberdelic media, its prospects, and challenges.

## Introduction

The theory that an alternative form of spiritual media can serve as an antidote to the maladies of alienated technological society and be key to solving global challenges may ring familiar to those acquainted with mid-twentieth-century psychedelic history. Back in the 1960s psychedelic advocates developed a vision of a society improved by the ingestion of psychoactives, which anthropologist Nicolas Langlitz terms a “political neurotheology” (Langlitz, 2011). Psychedelics, at the time, were viewed by many as transformational technologies that may help humanity realize its potential and climb further up the evolutionary ladder (Leary, 1993, 1998; Evans, 2022).

An updated version of the notion that technology may come to the rescue, enlighten and provide new horizons to a spiritually alienated humanity can be discerned in the concept of cyberdelics (Langford, 2019; Smith and Warner, 2022). Cyberdelics are technologies designed to produce altered states of consciousness (ASC) such as presence, awe, and transcendence, considered hallmarks of the mystical-type states afforded by psychedelics (Griffiths et al., 2006; Johnson et al., 2019). The term cyberdelic is a fusion of two words. The prefix “cyber” gestures towards cybernetics and the world of microchips, computer networks and digital media. The affix “delic” points at the mind-expanding world of psychedelic drugs.

A distinct product of the West Coast and the ‘Californian Ideology’ that flaunted a mix of experiential libertarianism, entrepreneurialism, and techno-utopianism (Barbrook and Cameron, 1996; Tvorun-Dunn, 2022), the concept of cyberdelics achieved some prominence in the 1980s and 1990s, at a time when the personal computer and internet revolutions were gathering steam. After receding from view in the 2000s, the concept has reemerged in recent years alongside a resurgence in psychedelic science and culture and a renewed interest in virtual reality technologies.

This perspective paper examines the cyberdelic idea: its cultural context, appeal, prospects, and challenges. By examining the story and ethos of cyberdelics as utopian, mind-manifesting technologies, it aims to illustrate the broader challenge of integrating mind-manifesting, psychedelic technologies within the context of contemporary high-octane, neo-liberal society. The endgame of this examination points to the inherent difficulty of modulating the nature of electronic (mind-manifesting) media within ingrained oppressive sociocultural conditions that favor dependent, compulsive patterns of media use. Within this context, cyberdelic media are in constant danger of being marginalized or repurposed to fit the values and norms of the surrounding society. The fate of these media therefore depends on their points of contact with broader sociocultural forces, including culture’s ability to allow for emergence of spaces of refuge from intense media intake, and its ability to imagine ways of being that are alternatives to the competitive, manipulation-oriented, consumerist matrix.

## A brief history of cyberdelia

The idea that digital technology may elevate human consciousness and assist humanity in realizing a loftier spiritual destiny may appear misplaced in the context of current discussions on the effects of media. The digital revolution, in particular its latest incarnations – the smartphone and social network revolutions – has been defamed in recent years. It is accused of wreaking havoc on human faculties of focus, concentration, empathy and connection, fomenting political polarization and unleashing an epidemic of mental illness (Carr, 2010; Turkle, 2011; Alter, 2017; Klimburg, 2017; Twenge, 2017a; Lanier, 2018; Zuboff, 2019; Feldstein, 2021). Digital media are furthermore posited to hook users in compulsory cycles of use, fueled by dependency on the regular secretion of neurotransmitter dopamine, facilitated through the use of smartphones and social media apps (Eyal, 2014; Lembke, 2021; Hartogsohn and Vudka, 2022).

Another dimension where digital technology’s effects are deemed to have been destructive is the domain of spirituality. Digital media are regularly described as robbing users of their sense of presence, bringing them into states of distraction, restlessness, fear of missing out (FOMO), anger, and envy that runs counter to spiritual ideals of presence, firmness and peace (Carr, 2010; Dossey, 2014; Fan et al., 2016; Faiola et al., 2018; Rosen et al., 2018; Rozgonjuk et al., 2020; Serrano, 2020; Hartogsohn, 2022).

Cyberdelic media present themselves as an alternative to the deleterious effects of contemporary digital media: media that cultivate harmony, awe, and presence rather than FOMO and dispersion, produce effects similar to those of the mystical-type experiences elicited by psychedelics, and are aligned with the aim of spiritual growth (Arnott, 2018; Glowacki et al., 2022; Smith and Warner, 2022). The cyberdelic project bases itself on the appealing suggestion that rather than fighting technology’s pernicious effects, technology could be redesigned to produce alternative, more favorable effects.

Cyberdelic history has roots in late 1960s and early 1970s, at a time when psychedelics were having their cultural heyday, and the personal computer and internet revolutions were still nascent. At the time, the psychedelic movement cultivated a complex and often ambivalent relationship to technology. On the one hand, the movement was strongly aligned with the back-to-the-land commune movement, which cultivated naturalistic, frugal, low-tech, and even anti-tech ideals (Miller, 1999; Fairfield and Miller, 2010). Simultaneously, the appearance of ‘mind-expanding’ psychochemical tools in the form of synthetic psychedelics seemed to hint at the possibility that other technologies might soon emerge, which would further empower individuals and societies. Openness to new technologies was salient in Stewart Brand’s iconic 1960s publication Whole Earth Catalogue, which endorsed for “access to tools” as a condition for personal and social growth (Turner, 2006). These tools included ancient and indigenous technologies next to high-tech ones. In the pages of the catalogue, writes Turner (2006, 200), “the moccasins and backpacks of those lighting out for the woods had bumped up against the calculators and technical manuals of Bay area engineers.”

It was against this backdrop that computers began to be imagined as a form of mind-altering technology. In 1968 media savant Marshall McLuhan cryptically proclaimed that “the computer is the LSD of the business world, “hinting perhaps at the enhanced cognitive terrains opened up by computation (McLuhan et al., 1968, 83). In 1972 the argument was made more explicit by Brand, who, in a Rolling Stone article about hackers, argued computers are the best news “since psychedelics” (Brand, 1972).

Over the next two decades, as digital technologies rose in prominence, psychedelics seemed to wane from the cultural horizon. Yet some digital visionaries held on to the hope that computers might hold the key to new mind-expanding breakthroughs. John Perry Barlow Grateful, a Grateful Dead lyricist turned digital ideologue, argued that virtual reality is the closest analogue to the psychedelic experience (Hagerty, 2000). VR pioneer Jaron Lanier propagated the idea that technology must be built to radiate “such beauty, fascination and depth that mankind will be seduced away from mass suicide” (Lanier, 2017, 299). Technologist Mark Pesce, originator of Virtual Reality Modeling Language (VRML) confessed to being inspired to the idea by a powerful LSD trip (Pesce, 2000; Hagerty, 2007). Virtual technologies, argued cyberdelic advocates, would allow us to simulate and inhabit the realities envisioned from within psychedelic altered states. Prominent icons of the psychedelic movement were embracing the notion that computers may soon provide similar services to those previously afforded by psychedelics. “The drugs of the future will be computers and the computers of the future will be drugs, “prophesized psychedelic icon Terence Mckenna (Cultural Frontiers in the Age of Information, 1994).^1^ Nineteen sixties psychologist turned LSD evangelist Timothy Leary also came onboard the cyberdelic trend by the 1990s. “Computers are the new LSD” proclaimed Leary, and updated his 1960s slogan “Turn on, Tune in, Drop out” to “Turn on, Boot up, Jack-in.” (Dery, 1996, 22).

Twentieth century cyberdelic culture peaked in the 1990s, alongside the internet revolution, and a small-scale psychedelic renaissance that emerged on the coasts of Goa and Ibiza, combining psychedelic compounds and electronic psytrance music designed to produce altered states of mind. However, the trend was short lived. While EDM and the internet revolution assumed a central cultural position, The VR winter of the 2000s, the fall of the Dot-com bubble, the corporatization of the web, the rise of digital surveillance, and other cultural factors such as the decline of the 1990s house/rave scene can all be surmised to have contributed to the waning of cyberdelic tide (Hartogsohn and Vudka, 2022).

Over the past two decades, a wave of techno-pessimism has all but completely reversed the socio-technical imaginaries around internet and information technologies. Digital technologies as such did not disappear from view, as can be recognized from the ascent of smartphones, social media and the cloud around this very time. Nevertheless, a radical shift has occurred in the ways such technologies are viewed and interpreted. Previously seen as technologies of individual empowerment aiding collective liberation, digital technologies came to be portrayed as inextricably enmeshed with the manufacture of addiction, corporate manipulation, political repression and a mental health epidemic (Morozov, 2011; Alter, 2017; Twenge, 2017a; Feldstein, 2021).

Rather than being seen as electronic psychedelics, digital technologies came to be viewed as analogous to narcotics and condemned as “electronic heroin” (Harsh, 2017; Phillips, 2017) and “digital cocaine” (Huddleston, 2016). “Virtual drugs” (Kardaras, 2017) were now considered addictive, debilitating, and harmful (Sutton, 2020; Hartogsohn and Vudka, 2022). The new view was actually a return to an older narcotic view of electronic media, which appears in the writing of heroin user and Beat writer William Burroughs and is explored by McLuhan in his “Notes on Burroughts.” According to Burroughs, the only possible escape from technology’s control is by regarding “our entire gadgetry as junk … apomorphine”(McLuhan, 1964, 518). The only way to be saved from the surveillance machine, Burroughs advises, is by shutting it off.

Then, in the mid-2010s something shifted again. Swept by the tides of a new psychedelic renaissance (Sessa, 2012; Brown, 2013; Pollan, 2018) and a virtual reality renaissance (Waldrop, 2017) – cyberdelics made their return.

## The current cyberdelic landscape

The cyberdelic renaissance consists of a plethora of digital technologies purporting to support personal and spiritual growth. On one end of the spectrum, one finds relatively familiar, even prosaic, technologies such as smartphone apps that guide users through meditation and breathing exercises, or assist psychedelic users in the integration of psychedelic experiences (Daudén Roquet and Sas, 2018; Noorani, 2021; Jablonsky, 2022). Other, more radical technologies for spiritual growth include neurofeedback devices that train users in controlling and calming their minds; brain-stimulation devices that purport to ‘down-regulate’ ego-centric brain areas in order to facilitate states of unity and increased fluidity; and computer-controlled light machines such as the Lucia No.3, which promise to induce states of wonder, awe, clarity and peace in users (Schwartzman et al., 2019; Wildman and Stockly, 2021). Much ‘Spirit Tech’ (Wildman and Stockly, 2021) is confined to research laboratories. However, some consumer products attempt to make such techno-spiritual wonders accessible to shoppers. Devices such as ‘Muse’ offer affordable neurofeedback hardware-software package that is promised to facilitate rapid and profound progress in users’ meditation practice. Another device, Xen by Neuvana promises to relieve stress, enhance focus and brighten mood using headphones that transmit micropulses directly to the vagus nerve (Muse Muse – Meditation Made Easy, 2021; Neuvana, 2022).

The field’s growth has also been fueled by the rising interest in VR technologies and in VR therapy in particular. Recent years have seen growing interest in the use of VR in the preparation and integration phases of psychedelic therapy, and possibly during psychedelic sessions (Aday et al., 2020; Gómez-Busto and Ortiz, 2020; Sekula et al., 2022). The two experiences have been described as overlapping, synergistic and complementary in their effects (Aday et al., 2020; Sekula et al., 2022). Strikingly, both psychedelic therapy and VR therapy are suggested as treatments for the same types of mental health problems including depression, anxiety, trauma and eating disorders (Gómez-Busto and Ortiz, 2020). With computer technologies again riding the crest of a psychedelic resurgence, it seems both movements are posed to comingle and interact in potentially synergistic ways.

Unsurprisingly, therefore, virtual reality technologies continue to hold a central place within the cyberdelic landscape. Some cyberdelic VR experiences promise to take users into a mystical Near Death Experience (Virtual Awakening, n.d.), others transport users into spectacularly colorful virtual worlds where sight and sound synesthetically mesh (Cyber Mushroom, 2022; Microdose, 2022), still other psychedelically inspired VR experiences like *Isness* purport to elicit empathy, ego dissolution and other Self-Transcendent Experiences (STEs) of the type induced by psychedelics by transporting users into a shared luminous virtual space where their beings are able to interact and even coalesce (Glowacki et al., 2020, 2022). In some cases, developers put these technologies to empirical tests that claim to demonstrate commensurability between their effects and those of psychedelics (Schwartzman et al., 2019; Glowacki et al., 2020, 2022).

Current efforts on the nexus of psychedelics and digital technologies are more subdued in their rhetoric compared to their 1980s counterparts. Nevertheless, the socio-cyberdelic imaginary (Schwarz-Plaschg, 2022) that advocates for the creation of mind-expanding technologies offers an alternative that challenges the domination of corporate-controlled, dependence-inducing forms of media. It can be recognized in the work of organizations such as The Cyberdelic Society which organizes “Cyberdelics Incubator” events where technologists and media artists present cyberdelic technologies. Such encounters aim to explore “how the targeted application of ancient and modern technologies can generate altered states leading to altered traits” (Cyberdelic Society Home, 2020). Cyberdelic society members point to the harms of contemporary digital media platforms and suggest that building better forms of technology might be the answer. “We’re critically at a stage in humanity where we need to get technology right so that it can help us rather than hinder us,” argues Smith (2019b). Others write manifestos calling for a new form of mind-altering technologies. The *Technodelic Manifesto*, written by Robin Arnott, founder and CEO of EntheoDigital – a company developing the cyberdelic meditation app Soundself, which converts user breath patterns into virtual visuals – adumbrates the contours of a technodelic media as media that goes against the grain of current digital technologies, by putting the emphasis on focus, presence, flow and eschewing goals and distractions. Arnott (2018) describes technodelic media as “fully immersive,” “doorways into the ineffable” (n.p.) and – similar to psychedelics – relying on the principles of set and setting, ritual and ceremony. Thus, while current cyberdelicists are careful to avoid utopian language, a proposition is made for a radical shift in the paradigm of human-machine relationships, one that will upend humanity’s debilitating dependency on incessant skinner-type electronic stimuli and allow electronic media to become doorways into the numinous.

## Discussion

Psychedelics remain a major source of inspiration for the emerging field of wholesome digital technologies, as the use of the “–delic” affix in terms like technodelic, cyberdelic and numendelic (the term used by Isness developer, David Glowacki) makes evidently clear. However, the high-tech nature of the cyberdelic project is sometimes linked with a certain unease. Cyberdelic Society co-founder Carl Hayden Smith, for instance, proposes a human centered vision of hyper-humanism as an alternative to the technology-centered vision of transhumanism promulgated by techno-evangelists (Smith, 2019b). Smith’s ‘hyperhumanism’ aspires to use technology to generate novel endogenous human capabilities, rather than gadget-based powers. “We do not want to wear technology. We want to become technology, “says Smith, quoting a tagline by CyborgNest, a company that develops technologies allowing users to expand their sensory horizons (CyborgNest | Human Augmentation Technologies and Sensory Enhancements, 2022). Cyborgnest’s products permit users to haptically connect with their family and network’s live heartbeats as measured by sensors, or with the earth’s magnetic field. Smith reports that after 6—8 weeks of wearing a belt that allows for magnetic sensing, a user gains the ability to autonomously detect north, even without putting on the belt. One reportedly learns to pick up on faint signals that were not noticeable before wearing the device, and a new sense is gained. The north-sense (Smith, 2019b). In a recent interview I held with him, Smith warned of being overdependent on sophisticated technologies that rely on complex and fragile supply-chains, a concern highlighted by the 2021—2022 global supply chains crisis (Gamio and Goodman, 2021).

Yet, beyond the question of supply chains, a more deeply felt suspicion of digital technology has been ingrained in contemporary society following revelations on the implication of such technologies in mass surveillance and election manipulation, and their disruptive economic, social and psychological effects (Carr, 2010; Morozov, 2011; Srnicek, 2016; Klimburg, 2017; Twenge, 2017b; Zuboff, 2019; Feldstein, 2021). Recent history has taught society to distrust the promises of digital utopia. Underlying this skepticism is a fundamental question: can digital technology be reformed or is it inherently pernicious?

Cyberdelic technologies offer hints that digital technology can be re-imagined. They provide evidence that computer technologies need not necessarily provoke FOMO, anxiety, and depression. Digital technologies can, under a different set of conditions, also produce experiences of awe, transcendence, empathy, and bliss. Still, the technological possibilities afforded by such media should not be conflated with social affordances, and herein lies the rub.

Social Shaping of Technology (SST) theory argues that technological design and use are inextricably meshed with their surrounding society and culture. Technology theorists have incessantly pointed to the myriad ways in which technologies tend to assume the mores, values and beliefs of their time and place (Bijker, 1997; MacKenzie and Wajcman, 1999; Pinch, 2009; Lievrouw and Livingstone, 2016; Greenfield, 2018). Examining the question of digital media through this lens, one may infer that digital media developed as they did – in a way that exacerbates addiction, consumerism and inequality – for a certain reason, namely, the distinct sociocultural and economic environments in which they evolved.

Interestingly, theories on the social shaping of technology resonate distinctly in one of the key concepts of psychedelic therapy and science: the idea of set and setting. Psychedelic compounds are regularly defined as mind-manifesting (from the Greek psyche-mind, delos-manifest) non-specific, amplifying agents whose effects depend, first and foremostly, on their context of use (Hartogsohn, 2017). These are, in other words, technologies defined by their susceptibility to environmental shaping. So much so that their effects change in nature each time they are used, corresponding with users’ mindsets and environment (Hartogsohn, 2015).

Tying together SST theory and the idea of set and setting, it is worth noting that some authors have identified information technology as mind-manifesting technologies, namely exceptionally versatile and context-dependent. “Computers are literally *psychedelic; that is, they manifest the mind, “*observes Erik Davis in his classic Techngnosis (Davis, 2015, 162). VR pioneer Jaron Lanier describes VR technology in a way that resonates common descriptions of psychedelics as magnifiers of consciousness (Schneider, 1967; Lee and Shlain, 1992; Grof, 2008; Strassman et al., 2008; Hartogsohn, 2018), arguing that “virtual reality will test us. It will amplify our character more than other media ever have” (Lanier, 2017, 1), and that the technology is “capable of amplifying both the best and worst in people” (Lanier, 2017, 142). Cyberdelics proponents also regularly point to the essential relevance of the psychedelic set and setting principle for cyberdelic technologies, which, they argue, are synergistic with the use of ritual and integration (Lanier, 2017, 153; Arnott, 2018; Smith and Warner, 2022).

Such proclamations regarding the context-dependency of digital technology provide valuable clues for the reasons digital technologies developed along narcotic, not cyberdelic, lines. As Yuval Noah Harrari opined regarding disruptive technologies of human-enhancement “If you have an arms race with these technologies then it’s very clear what kind of human abilities are going to optimized: it will be things like intelligence, it will be things like discipline … but other things like compassion, like artistic appreciation, like spirituality – these are things that are not very high on the list of armies and corporations. So the attempt to upgrade humans might actually result in downgraded humans.”(Smith, 2019a).

Digital media’s narcotic affordances of alienation and numbing should therefore be seen in the context in which they emerged. They fit the conditions of life in a competitive, hyper-individualist, neo-liberal culture characterized by boredom, a meaning crisis, and rising costs of living (Alexander, 2010; Duménil et al., 2011; Vervaeke et al., 2017; Mastropietro and Vervaeke, 2020). Their repetitive, addictive nature serves to dull the pain and anxiety of contemporary life while providing easy distractions. There is a reason why individuals in contemporary society find it easier to make room in their lives for smartphones and social media apps rather than to neurofeedback devices or awe-inspiring VR experience. The stimulating electronic dopamine-rush appears better suited to the pace of 21st century capitalist civilization where time is fragmented and scarce (Wajcman, 2016). Displacing the everyday dominance of information glut and addictive variable-reinforcement schedules with awe, unity and transcendence appears unlikely within the logics of a system prioritizing manipulation, efficiency, profit, and control. Media theorist Corey Anton suggests a useful distinction between Apollonian tight drugs (such as Ritalin, caffeine and Prozac) which facilitate ordered, productive behavior that is in line with accepted social norms, and Dionysian loose drugs (such as psychedelics), which more radically subvert perception (Anton, 2012). In light of Anton’s distinction, one is advised to remember that despite talk of a psychedelic renaissance, use of psychedelics is expected to remain negligible in volume in comparison to use of stimulants such as coffee, nicotine and amphetamines and depressants like alcohol and benzodiazepines. If computer technologies are mind-manifesting as argued by Davis, it makes sense that in the context of contemporary society they would naturally evolve towards the tight variety, rather than the loose one.^2^

Is the quest for self-transforming and potentially society-transforming cyberdelic technologies inherently futile as long as society retains its current form? Folded in such discussions is one of the key questions in the theory of technology. Does technology shape society or does society shape technology? (Smith and Marx, 1994) While mid-twentieth-century psychedelic evangelists promised psychedelics will change society (echoing the popularity of mid-twentieth-century media-theories that awarded technology primacy over society; Havelock and Havelock, 1963; Leary, 1998; McLuhan, 2003; Innis, 2007, 2008), contemporary discussions about psychedelics have been characterized by socially constructivist sensitivities that echo the constructivist turn in the social sciences, including the social studies of science and technology (Hacking and Hacking, 1999; MacKenzie and Wajcman, 1999; Hardon and Sanabria, 2017; Hartogsohn, 2020; Pace and Devenot, 2021). Eventually, one inevitably returns to the recognition that the relationship between technology and society is inherently complex: bi-lateral, co-produced (Jasanoff, 2004), networked (Callon, 1984; Latour, 1993, 2007) and even post-structural (Brunner, 2014). Mind-manifesting technologies take part in these complex dynamics with arguably even greater intensity than other categories of technology,

The challenges of cyberdelics therefore appear to mirror current conversations within the psychedelic humanities about the moral and political implications of the drugs (Hartogsohn, 2020; Timmermann et al., 2020; Langlitz et al., 2021; Pace and Devenot, 2021). Medicalization was originally judged positively by many in psychedelic space and viewed as a route to greater social legitimization. Current discussions are more skeptical, highlighting the importance of cultural context in which a psychedelic resurgence takes place and regarding the merits of such a resurgence as contingent on the beliefs, models and relationships that define it (Langlitz et al., 2021; Pace and Devenot, 2021; Hauskeller et al., 2022; Schwarz-Plaschg, 2022).

The context of a cyberdelic resurgence is similarly crucial, though for different reasons. Products of deliberate engineered design, digital drugs are significantly more specific in their effects than the unruly psychedelics. The question here is whether a swing away from narcotic media and towards cyberdelic media is realistic given the socio-economic and cultural context of contemporary media use, and whether such media may earn their place in the competitive attention economy. While neo-liberal, free-market society continues to exert unabated pressures over a squeezed middle-class (Piketty, 2014), the COVID-19 pandemic has also led to a much discussed great resignation (Jiskrova, 2022; Sull et al., 2022). A turn in societal relationship to digital media is recognizable in the growing interest in ideas of mindful technology design (Neumann, 2016; Langford, 2019; Martin, 2021) and practices of digital minimalism (Newport, 2019). Meanwhile a diverse community of ‘consciousness hackers’ is keen on the creation and exploration of alternative, ‘holistic’ forms of media (Wildman and Stockly, 2021). Cyberdelics therefore may be able to carve out a niche of digital mind-expansion. Furthermore, while a complete reversal of trends appears a remote possibility, it is important to note that a social tendency towards the tight (narcotic) variety of digital media does not preclude a concurrent ascent of loose (cyberdelic) media. A person may, for instance, use a VR headset to have occasional cyberdelic experiences, and still compulsively use smartphones and social media much in the same way that users of psychedelics do not necessarily give up use of stimulants such as coffee or ritalin.

Like psychedelic neuro-theology, the cyberdelic project can be viewed as an attempt to invert the logic of media in order to foster alternative states of mind and shift the human condition characteristic of its era. For now, Cyberdelics remain an exotic form of alternative media which augurs exciting possibilities of undetermined prospects. Whether the project will ultimately come to be regarded as visionary or naïve will depend on the ways in which these mind-manifesting technologies come into contact with society’s cultural imagination and culture’s ability to imagine and support alternative ways of being outside the dopamine-addled consumerist matrix.

## Data availability statement

The original contributions presented in the study are included in the article/supplementary material, further inquiries can be directed to the corresponding author.

## Author contributions

The author confirms being the sole contributor of this work and has approved it for publication.

## Conflict of interest

The author declares that the research was conducted in the absence of any commercial or financial relationships that could be construed as a potential conflict of interest.

## Publisher’s note

All claims expressed in this article are solely those of the authors and do not necessarily represent those of their affiliated organizations, or those of the publisher, the editors and the reviewers. Any product that may be evaluated in this article, or claim that may be made by its manufacturer, is not guaranteed or endorsed by the publisher.

## Footnotes

^1^The phrasing of McKenna’s quip, given at the end of a Q&A session, were more meandering, but the quote above is the one perennially in use over the web.

^2^Importantly, a tendency towards the tight (narcotic) variety of digital media does not preclude a possible ascent of loose (cyberdelic) media. A person may, for instance, use a VR headset to have occasional cyberdelic exeprienes, and still compulsively use social media, email etc.
